# Diagnosis acceptance, masking, and perceived benefits and challenges in adults with ADHD and ASD: associations with quality of life

**DOI:** 10.3389/fpsyt.2025.1668780

**Published:** 2025-10-15

**Authors:** Paula Wurth, Anselm BM Fuermaier, Anne Hege Strand, Lisa B. Thorell

**Affiliations:** ^1^ Division of Psychology, Department of Clinical Neuroscience, Karolinska Institute, Stockholm, Sweden; ^2^ Department of Clinical and Developmental Neuropsychology, University of Groningen, Groningen, Netherlands; ^3^ The Fafo Institute for Labour and Social Research, Oslo, Norway

**Keywords:** neurodevelopmental disorders, ADHD, ASD, quality of life, masking, diagnosis acceptance, benefits, and challenges

## Abstract

**Objectives:**

Research has increasingly focused on neurodivergent individuals’ acceptance of their diagnosis and the extent to which they engage in masking behaviors. However, there is a lack of large-scale quantitative investigations. This study, therefore, examined how young adults with attention deficit hyperactivity disorder (ADHD) and/or autism spectrum disorders (ASD) perceive and relate to their diagnosis in terms of diagnosis agreement, diagnosis liking, masking, perceived benefits/challenges, and how these constructs are related to one another and to quality of life.

**Methods:**

The sample included adults with ADHD (*n* = 803), ASD (*n* = 158), or both ADHD and ASD (*n* = 95). Participants (aged 18-35; 79% females) completed an online survey assessing diagnosis acceptance, perceived benefits and challenges, masking, and quality of life.

**Results:**

The participants reported high agreement with their diagnosis (95%), but fewer liked their diagnosis (29%), and masking behavior varied by context. Participants reported experiencing both benefits (e.g., “seeing things my own way” and “drive to find things out”) and challenges (e.g., “mentally exhausting” and “being misunderstood”) related to their diagnosis. Quality of life was associated with all other variables, but most strongly associated with perceived benefits.

**Conclusions:**

This study shows that adults with ADHD and/or ASD generally agree with their diagnosis, but the extent to which they like their diagnosis varies. Masking is commonly reported and depends on the context. Perceiving benefits was the variable most strongly associated with quality of life, highlighting the importance of clinical approaches promoting strength-based perspectives rather than a strict deficit approach.

## 1 Introduction

Neurodevelopmental disorders such as attention-deficit hyperactivity disorder (ADHD) and autism spectrum disorder (ASD) are characterized by neurocognitive deficits (1), which significantly impact the individual’s daily life functioning. Systematic reviews and meta-analyses have also shown that, compared to healthy controls, individuals with ASD (2) or ADHD (3) often report lower quality of life. Given that neurodevelopmental disorders are increasingly recognized as lifelong conditions that cannot be “cured”, enhancing quality of life represents a central and clinically meaningful goal for ADHD interventions. Notably, prior research indicates that quality of life in ADHD is often unaffected by medication, comorbid psychopathology, or psychosocial treatment (4). However, factors related to diagnosis self-perception are associated with well-being (5). Over the past few decades, increasing attention has also been paid to the neurodiversity movement and minority stress models in understanding these conditions (6–8). These approaches emphasize the need to complement the traditional focus on challenges that individuals with ADHD or ASD face, with an appreciation of their strengths, how to best promote the benefits of neurodivergent individuals, and the integration of being neurodiverse into the individuals’ identity (9–12). However, while valuable, most research on ADHD and ASD remains deficit-focused, and studies exploring positive aspects of these conditions have often used small samples and qualitative methods (13–15). It has also been argued (16) that more studies are needed to examine how factors such as diagnosis acceptance and perceived benefits of having a neurodevelopmental disorder are related to real-life outcomes such as quality of life. The overall aim of the present study was, therefore, to examine how young adults with ADHD and/or ASD experience and relate to their diagnosis and to what extent this is associated with quality of life.

Diagnosis acceptance (i.e., whether people agree with or like the diagnosis that they have received) has recently emerged as a salient theme in qualitative research on individuals with ASD (17, 18) and ADHD (19), and a positive diagnostic identity has been associated with better mental outcomes and higher quality of life in ASD (17, 20–22). In fact, the diagnostic process itself may yield emotional benefits such as validation, relief, and enhanced belonging (19, 23). Whether people agree with or like their diagnostic label can also influence the extent to which they seek and engage in treatment (24).

Individuals with neurodevelopmental conditions often report perceived diagnosis-related benefits, such as honesty and being able to hyperfocus (13), as well as creativity, resilience, and socio-affective skills (14, 16). While such factors may reflect personal strengths rather than diagnostic features, their perceived connection to one’s neurodevelopmental diagnosis contributes positively to their sense of belonging to a group (25). However, these same aspects can also be perceived as burdensome or challenging depending on the context (13), or coexist alongside diagnosis benefits (15, 18). Most studies in this area of research have been qualitative. Thus, there is a need for large-scale quantitative studies examining the proportion of individuals who accept their diagnosis and how many perceive benefits. Additionally, it is unknown to what extent acceptance is related to benefits and challenges, and how these three constructs are related to quality of life.

Another important aspect is the extent to which individuals with neurodevelopmental disorders engage in masking behavior. This has been defined as a key component of social camouflaging and refers to the concealment of autistic traits and the adoption of alternate personas in social situations (26). Masking has been well-documented among individuals with ASD (27, 28). Although less studied, emerging evidence suggests that individuals with ADHD also engage in masking (29). While masking may have short-term social advantages, it has been repeatedly linked to long-term costs such as reduced quality of life and increased mental health difficulties among individuals with ASD (17, 28, 30). Research has also shown that masking often emerges in response to societal stigma (31, 32). Recent findings suggest that masking varies across contexts, with individuals with ASD reporting that they use camouflaging less in environments they perceive as accepting or similar in communication style (33). Despite this, few studies have investigated masking in different settings such as schools, workplaces, within the family, or in interactions with individuals who share the same diagnosis. More studies are also needed to explore whether masking differs across diagnostic categories and how it relates to quality of life.

Although no theoretical framework fits perfectly with this study, it is relevant to Acceptance and Commitment Therapy (ACT), a framework widely applied to people with neurodevelopmental conditions (34, 35). First, agreement with and liking one’s diagnosis reflect acceptance, one of the six central processes in ACT. Second, perceiving diagnosis-related benefits maps onto values-based living, another key ACT process. Third, masking can be understood as a form of experiential avoidance, commonly linked to adverse psychological outcomes (36). Promoting these processes via ACT is linked to many psychological outcomes, including quality of life (37).

The overall aim of the present study was to use a large-scale, quantitative approach to investigate how young adults with ADHD and/or ASD experience and relate to their diagnosis and to what extent this is associated with quality of life. More specifically, we aimed to address the following research questions:

1. To what extent do individuals with ADHD and/or ASD agree with and like their diagnosis?
2. To what extent do individuals with ADHD and/or ASD engage in masking behaviors across three different contexts (i.e., school/work, family, neurodivergent peers)?
3. What are the most commonly reported diagnosis benefits and challenges among individuals with ADHD and/or ASD?
4. How are the three factors mentioned above (i.e., agreement with one’s diagnosis, diagnosis liking, masking behaviors, and number of perceived benefits and challenges) associated with quality of life?

## 2 Methods

### 2.1 Participants

This study included 1,056 Norwegian young adults (79% females), of whom 803 had been diagnosed with ADHD (83% females), 158 with ASD (62% females), and 95 (70% females) with both ADHD and ASD. The target group was young adults aged 18 to 35 years, with a mean age of 28 years (*SD* = 5.47, range 18-35). They had received their diagnosis at an average of 6 years (median = 3 years) prior to the study. The most commonly reported psychiatric comorbidities were anxiety (57%), depression (59%), anxiety and depression combined (46%), and burnout (38%). Regarding medication use, 79% of participants reported current use of medication. Full demographic data can be found in the **Supplementary Table 1**.

### 2.2 Procedure

The participants were recruited via patient organizations and word-of-mouth using a self-selection, river-sampling approach. They were asked to complete an anonymous online survey that took approximately 20 minutes. The survey was created, piloted, and reviewed with people with lived experiences to ensure clarity and relevance. Only minor language edits to enhance clarity for a few items were needed after the pilot study. Data was collected via the platform QuenchTec from December 2022 to March 2023. All participants provided informed consent, and ethical consent was provided by the Norwegian Agency for Shared Services in Education and Research (#102670).

### 2.3 Measures

#### 2.3.1 Diagnosis liking

Liking one’s diagnosis was measured using the following two items: “I like being autistic” and “I like having ADHD.” Each participant answered only the item relevant to his/her own diagnosis. Both items were rated on a 5-point scale ranging from 1 (strongly disagree) to 5 (strongly agree).

#### 2.3.2 Diagnosis agreement

Diagnosis agreement was assessed using an item “Do you agree with the diagnosis/diagnoses you have received?” and responses were made on a 3-point scale: 1 (yes, agree), 2 (neither agree nor disagree), and 3 (no, disagree).

#### 2.3.3 Perceived benefits and challenges

The items used to assess perceived benefits and challenges were developed through a combination of researcher expertise, input from patient organizations, and feedback from a pilot study. In the pilot phase, young neurodivergent individuals tested the questionnaire and provided comments on the items’ relevance and clarity. This co-creative process helped refine the list of indicators to reflect aspects considered meaningful by both the target population and the research team. Each item was rated on a binary scale (yes/no), and the following benefits were included: 1) seeing things my own way, 2) sense of justice, 3) community with others, 4) attention to details, 5) honesty, 6) distinctive features, 7) positive energy, and 8) drive to find things out. The challenges listed were 1) physically tiring, 2) mentally exhausting, 3) differential treatment, 4) being misunderstood, 5) being disrespected, and 6) criticism of things related to the diagnosis. Participants could also select the option “Nothing” if they did not experience any of the mentioned challenges or benefits. In addition to investigating the prevalence of each benefit and each challenge, we also calculated sum scores for the number of perceived benefits (ranging from 0 to 8) and challenges (ranging from 0 to 6).

#### 2.3.4 Masking behaviors

Masking behaviors were measured using the question: “I feel that I have to mask my personality when I am…” ending with three different scenarios: 1) “at school or work”, 2) with people in my family”, and 3) “with others with my diagnosis”. These items were measured on a 5-point scale ranging from 1 (Completely disagree) to 5 (Completely agree), with the additional response “not relevant”, coded as a missing value.

#### 2.3.5 Quality of Life

Quality of life was assessed using a 3-item scale. The items captured life satisfaction (“All in all, how satisfied are you with your life at the moment?”), the feeling of doing something meaningful (“All in all, to what extent do you feel that what you are doing in life is meaningful?”), and life satisfaction five years from now (“All in all, how satisfied do you think you will be in your life five years from now?”). Responses were given on an 11-point scale from 0 (“Not satisfied at all” or “Not meaningful”) to 10 (“Very satisfied” or “Very meaningful”). The three items showed good internal consistency (Cronbach’s α = .87, range.85 -.90 for the three subgroups).

### 2.4 Statistical analyses

Statistical analyses were conducted using SPSS (version 29), with RStudio (version 2025.12.1) being used for data visualization. Outliers were only found for one of the variables (“Masking at school/work”), and the values for all participants scoring 1 on this item (n = 64) were adjusted in line with the Outlier Labeling Rule (38). Descriptive statistics were computed to explore overall patterns in outcome variables. To examine group differences in outcome variables across diagnostic groups and sex, group comparisons and potential interaction effects were analyzed using one-way ANOVAs. Regarding the assumptions of ANOVA, we tested for outliers (see above), checked for homogeneity of variances using Levene’s test, and tested for normality of the residuals. For variables showing non-homogeneity of variances, we used Welch’s ANOVAs to investigate main effects, and Games-Howell tests for *post hoc* comparisons. All variables showed non-normality of the residuals. However, as even small deviances in normality can be detected with sample sizes as big as the present study, we report results from the ANOVAs as the main analyses. In addition, we re-ran the analysis for diagnostic group differences using the Krusal-Wallis test to determine if the results changed when using a non-parametric test. Sex differences were investigated using independent samples t-tests. To examine group differences for diagnosis benefits and challenges (coded as binary yes/no variables), we conducted chi-square tests. First, we tested for overall group differences using 3x2 contingency tables. Then, we performed *post-hoc* pairwise comparisons for significant effect measures from step one using 2x2 tables, comparing each diagnostic group with the others (ADHD vs ASD, ADHD vs ADHD+ASD, and ASD vs ADHD+ASD). We controlled for multiple comparisons using the Holm-Bonferroni correction in all analyses investigating group differences.

Pearson correlations were used to investigate bivariate correlations between diagnosis acceptance, masking behaviors, the number of perceived benefits and challenges, and quality of life. A linear regression analysis was conducted to assess how diagnosis liking, masking behaviors, and perceived benefits and challenges were uniquely associated with quality of life. This regression analysis was also re-run, entering age, sex, and time when diagnosis as control variables in step 1 and the remaining variables in step 2. The variance inflation factor (VIF) values for all independent variables were below 2 (Tolerance > 0.5), indicating that multicollinearity was not a concern. A significant threshold of p <.05 was used for all analyses.

There were no missing data for the variables “diagnosis liking” and “quality of life”. For masking within the family, 28 (2.7%) individuals had missing data; the corresponding number was 9 (0.9%) for masking at school/work. For masking with neurodivergent peers, 92 (8.7%) individuals had missing data, of which most of them reported that this item was not relevant because they never or seldom met people with the same diagnosis. In total, the sample sizes varied between 755–803 for the ADHD group, 125–158 for the ASD group, and 84–95 for the ADHD+ASD group. As only people with no missing variables can be included in a regression, this resulted in a sample size of 940 in this analysis. Pair-wise deletion was used for all other analyses.

## 3 Results

### 3.1 Agreement with diagnosis and liking diagnosis

**Figure 1:**
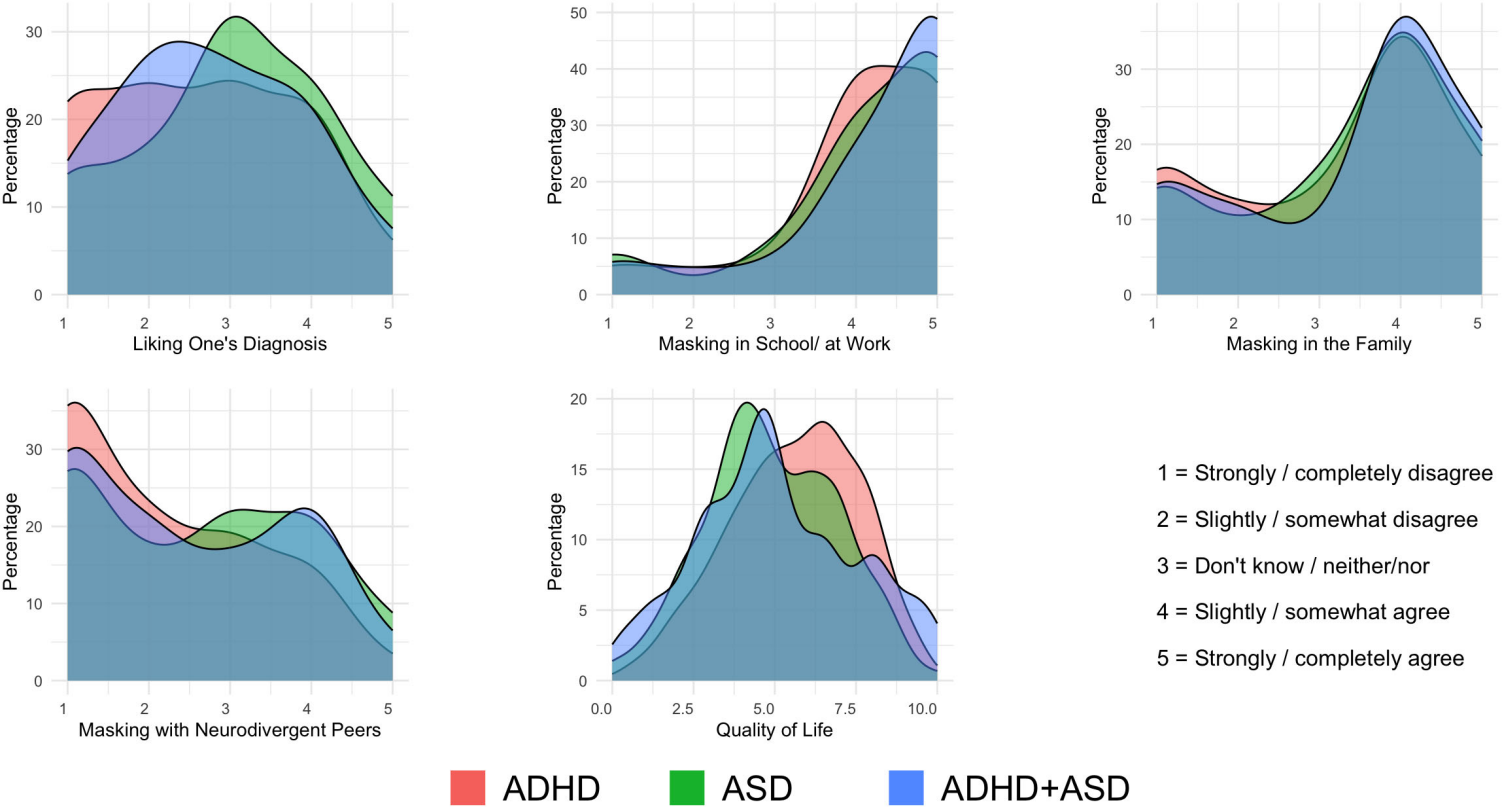
Density plots of diagnosis liking, masking behaviors, and quality of life across diagnosis groups. Density plots by diagnostic groups for each outcome variable. The x-axes include item response options for liking one’s diagnosis and for masking items. Benefits and challenges show the total number of items clicked. Quality of life shows the mean score. The y-axes show smoothed percentages using kernel density estimation. Fill colors indicate diagnostic groups.

The results revealed very little variance for the variable agreement with diagnosis, with as many as 95% of the participants answering “Yes, agree” to this question. Due to this ceiling effect, this variable was excluded from subsequent analyses. **Figure 1** displays density plots for the variables related to liking and masking. A considerable variation in the scores was found regarding the extent to which the participants liked their diagnosis, with 46% responding “strongly disagree” or “slightly disagree”, 25% responding “neither agree nor disagree”, and 29% responding “somewhat agree” or “strongly agree”.

As shown in **Table 1**, significant group differences were found for diagnosis liking. *Post hoc* analyses revealed that individuals with ASD scored higher than the ADHD group. This group difference in the ANOVA remained significant when using the non-parametric Krusal-Wallis, *H* ≥ 13.64, both *p* <.001.

**Table 1:** Results of ANOVAs investigating group differences.

	ADHD group ^a^ (1)	ASD group ^a^ (2)	ADHD+ASD group ^a^ (3)			
	M (SD)	M (SD)	M (SD)	*F*-value	η^2^	*Post hoc*
Liking^b^	2.60 (1.21)	3.01 (1.20)	2.76 (1.14)	7.83***	.015	2 > 1
Masking						
School/work	4.13 (0.96)	4.17 (1.02)	4.29 (1.03)	1.03	.002	n.s.
Family members	3.24 (1.39)	3.37 (1.35)	3.41 (1.39)	1.05	.002	n.s.
Neurodivergent peers^b^	2.15 (1.19)	2.59 (1.35)	2.45 (1.32)	7.13**	.017	2 > 1
Quality of Life^b^	5.62 (1.98)	4.91 (2.03)	5.06 (2.46)	9.66***	.019	1 > 2

**p <.01; ***p <.001.

a Sample sizes: n = 755–803 for the ADHD group; n = 125–158 for the ASD group; n = 84–95 for the ADHD+ASD group.

b Because the assumption of heterogeneity was violated for these variables, we used the Welch test for investigating main effects and the Games-Howell test for *post hoc* comparisons.

### 3.2 Masking

The three types of masking exhibited very different distributions, with a relatively small percentage of individuals with ADHD and/or ASD (i.e., 21% scoring ≥ 4.0) masking their personalities when meeting others with the same diagnosis. However, a substantial proportion of the participants (i.e., 57% scoring ≥ 4.0) reported that they engage in making within the family, and an even larger proportion engage in masking in school/at work (i.e., 83% scoring ≥ 4.0).

Significant group differences were also found for masking with neurodivergent peers. *Post hoc* analyses showed that individuals with ASD scored higher than the ADHD group for masking among others with the same diagnosis. No significant group differences were found for the other types of masking. The significant effect in the ANOVA was confirmed by the Kruskal-Wallis test, *H* ≥ 13.64, both *p* <.001. Only two out of three masking scenarios exhibited significant effects for sex, with medium effect sizes for masking in school/at work and small effects for masking with the family (see **Supplementary Table 2**).

### 3.3 Benefits and challenges with diagnosis

The average amount of reported benefits was 3.63 (*SD* = 2.03, range 0-8), with the most commonly endorsed benefits being “seeing things my own way” (65%) and “drive to find things out” (62%). The least common benefits were “positive energy” (25%) and “community with others with the diagnosis” (28%). For benefits, 6.6% of the participants reported no benefits (ADHD: 6.2%; ASD: 10.1%; ADHD+ASD: 4.2%). The corresponding numbers for challenges were 0.5% (ADHD: 0.5%; ASD: 1.9%; ADHD+ASD: 0%).”

The average amount of challenges mentioned was 3.68 (*SD* = 1.51, range 0-6) aspects that they disliked about their diagnoses. “Mental exhaustion” (94%) and “being misunderstood” (84%) were the most frequently endorsed challenges, while “being disrespected” (40%) and “differential treatment” (33%) were the fewest reported ones.

**Figure 2:**
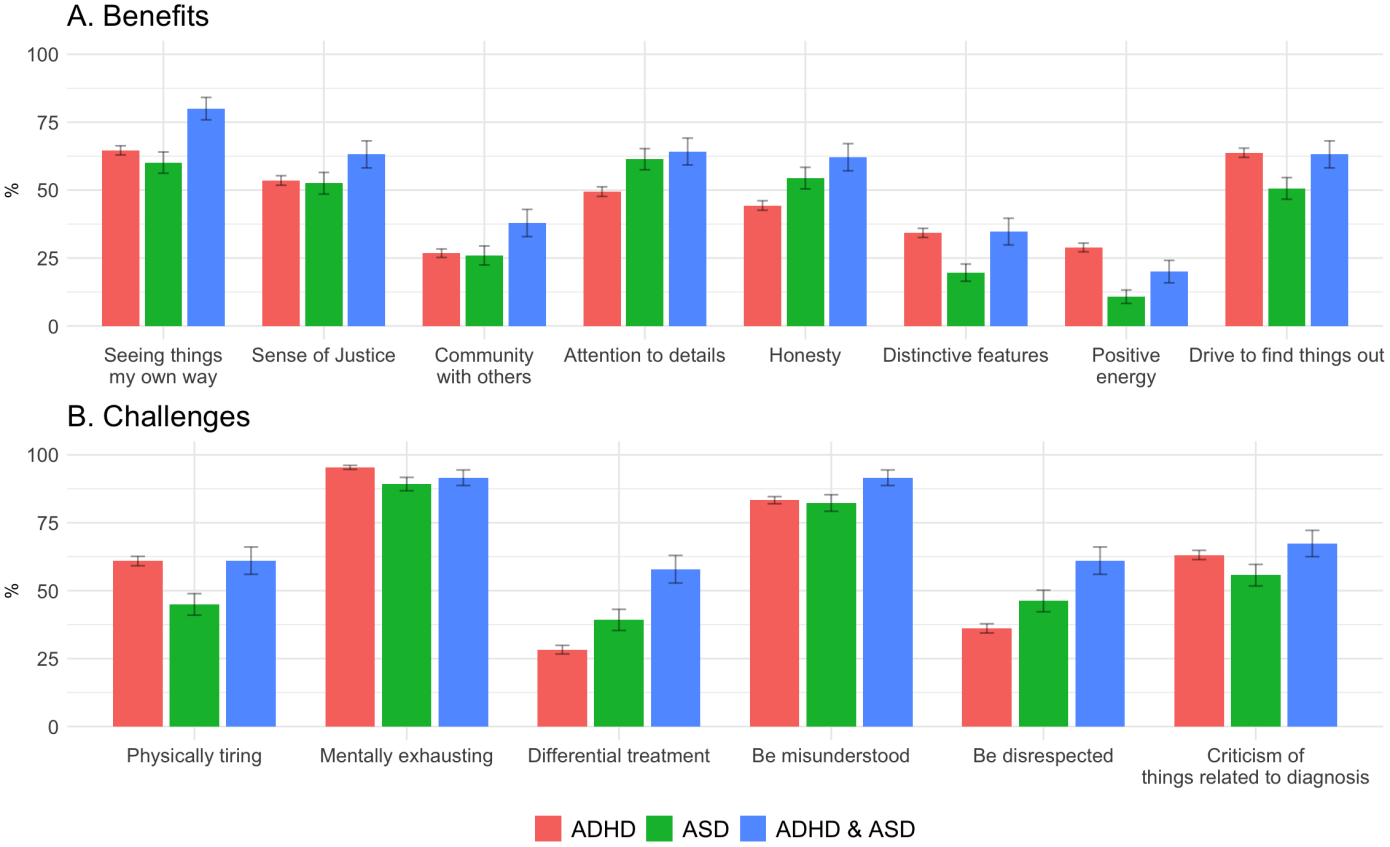
Diagnosis benefits **(A)** and challenges **(B)** endorsements across groups.

**Figure 2** and **Supplementary Table 3** display group differences for benefits and challenges. Regarding benefits, participants in the ADHD+ASD group more often than both other groups endorsed “seeing things my own way” as a benefit. Both the ASD and ADHD+ASD groups endorsed “attention to detail” and “honesty” more frequently than the ADHD group. In addition, individuals with ADHD or ADHD+ASD more often reported experiencing “distinctive features” compared to those with ASD. The ADHD group also more often endorsed “positive energy” and “drive to find things out” compared to the ASD group.

Regarding challenges, the ADHD and ADHD+ASD groups more frequently reported finding their diagnosis “physically tiring” than the ASD group, while the ADHD group reported “mentally exhausting” more often than the ASD group. “Differential treatment” and “being disrespected” were most frequently endorsed by the ADHD+ASD group, followed by ASD, and then ADHD). In summary, there was a tendency for the ADHD+ASD group to more often endorse both challenges and benefits. However, it should also be noted that in terms of effect sizes, most diagnostic group differences were small (χ^2^ (2, *N* = 1,056) < 25.10, Cramér’s *V* <.15), with medium-sized effects (i.e., [χ^2^ (2, *N* = 1,056] < 37.70, Cramér’s *V* = .19, *p* <.001) only being found for “differential treatment”. A small effect of sex could be found for the total number of challenges (**Supplementary Table 3**).

### 3.4 Association with quality of life

Overall, the participants’ mean quality of life was 5.49 (*SD* = 2.04) on an 11-point scale. The main group difference was significant, with *post hoc* analyses showing that individuals with ADHD reported a significantly higher quality of life than the ASD group (**Figure 1** and **Table 1**). The group difference remained significant when using the non-parametric Kruskal-Wallis, *H* = 20.45, *p* <.001.

**Figure 3:**
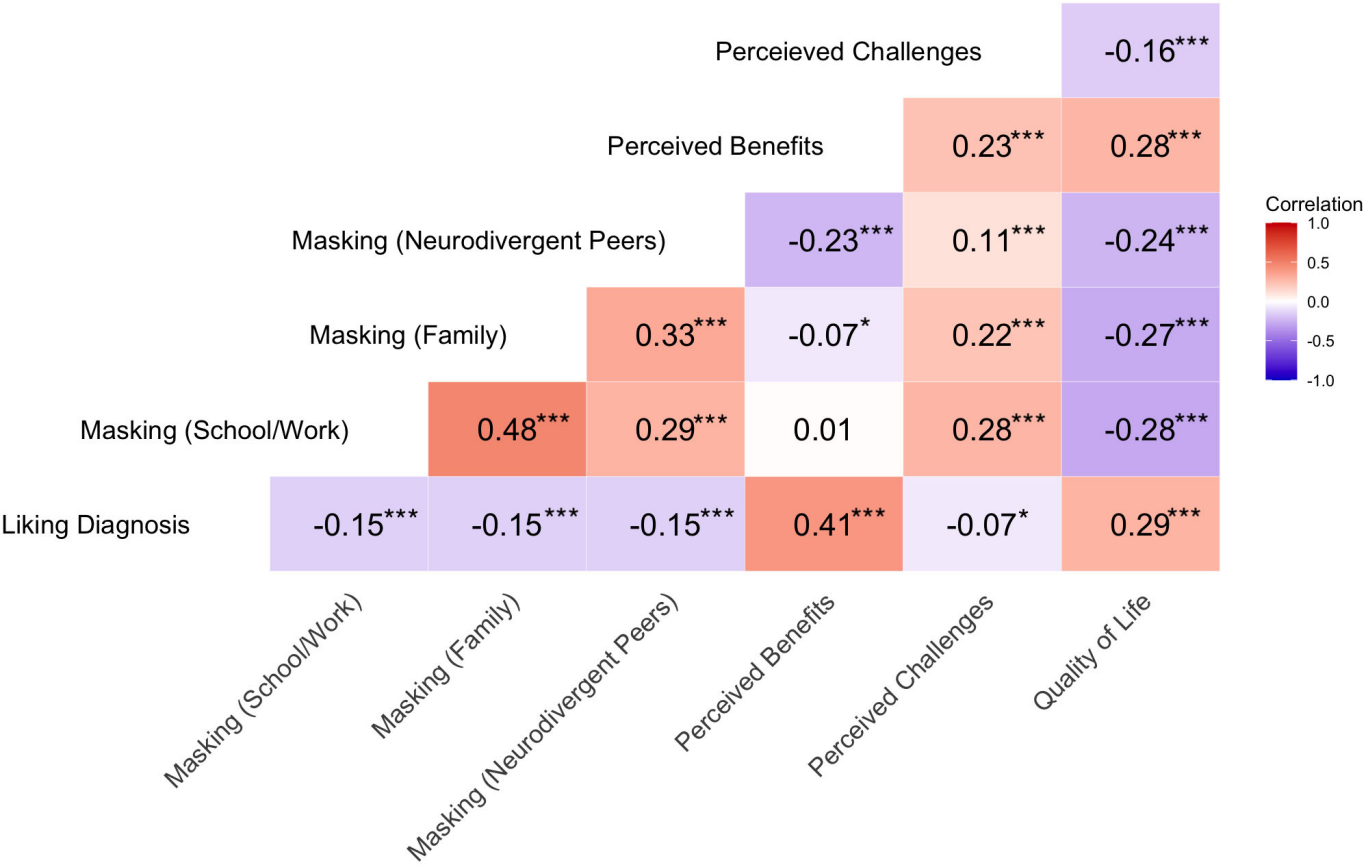
Correlation heatmap. This heatmap displays pairwise Pearson correlation coefficients between variables. *p <.05, ***p <.001.

Regarding associations between quality of life and the other variables, the results (see **Figure 3**), showed that quality of life was positively associated with liking one’s diagnosis (*r* = .29) and perceived number of benefits (*r* = .28). In contrast, quality of life was negatively associated with all three masking items (school/work: *r* = -.27; family: *r* = -.27; neurodivergent peers: *r* = -.24) and perceived number of challenges (*r* = -.16). When investigating specific benefits and challenges (not shown in **Figure 3**), all of them showed significant associations with quality of life. For benefits, the strongest associations were found for “positive energy” (*r* = .28) and “drive to find things out” (*r* = .22). For challenges, the strongest associations were found for “differential treatment” and “being disrespected” (both *rs* = -.13).

Next, we conducted a linear regression to assess the unique contribution of each independent variable while controlling for shared variance among these variables. Results showed that all variables were significant, *F*(6, 939) = 43.02, *p* <.001, with beta-values ranging from -.08 for “masking with neurodivergent peers” to.22 for “perceived benefits”. In total, the model explained 22% of the variance in quality of life. The results did not change when including sex, age, and time since receiving the diagnosis as control variables in step 1, and these variables only explained 0.3% of the variance in quality of life (see **Supplementary Table 4**).

In addition to associations with quality of life, interrelations between all variables were also assessed (**Figure 3**). Of note was that liking one’s diagnosis was moderately related to the number of perceived benefits (*r* = .41), but only weakly to the number of challenges (*r* = .07). In addition, the three masking contexts – school/work, family, and neurodivergent peers – were weakly to moderately correlated (between *r* = .29 and.48) and masking among neurodivergent peers (r = -.23) was most strongly associated with perceived benefits. In contrast, perceived challenges were positively correlated with masking in school/at work (*r* = .28) and family (*r* = .22), while the correlation was weaker with neurodivergent peer masking (*r* = .11).

### 3.4 Sensitivity analyses

Finally, we conducted some sensitivity analyses to determine if the results changed when investigating the effects of medication use and comorbidities. The results showed that individuals with and without medication did not differ on any of the variables, all *t*s < 1.27. The exception was quality of life (*t* = 2.00), but this effect did not remain significant when controlling for multiple comparisons. Regarding comorbidities, individuals with comorbidities (*t*s > 2.97, *p* <.01) reported significantly lower levels of diagnosis liking and quality of life, but higher levels for the three types of masking and disadvantages, compared with those without a comorbid disorder. The group difference for benefits was not significant, and the same results were found when comparing individuals with or without a mood disorder (*t*s > 2.97, *p* <.01. All effect sizes were small, except for the group difference for quality of life, which showed a medium-sized effect (*d* = .57).

## 4 Discussion

This study aimed to examine patterns of diagnosis acceptance (i.e., agreeing with and liking one’s diagnosis), perceived benefits and challenges, masking behaviors, and quality of life using a large sample of 1,056 adults with ADHD and/or ASD. The results showed that most participants agreed with their diagnosis, whereas more variability was found for liking one’s diagnosis. Masking appeared to be context-dependent, with the highest levels reported in school and family settings. Second, while participants endorsed both benefits and challenges related to their diagnoses, challenges such as mental exhaustion were nearly universal. Third, quality of life was associated with liking one’s diagnosis, perceived benefits and challenges, and masking behaviors, with perceived benefits showing the strongest association.

Diagnosis acceptance has often been conceptualized as diagnosis agreement and/or diagnosis liking in previous studies (39, 40). In our study, both these aspects were examined as part of the first research question. The results showed that almost all participants agreed with their diagnosis. However, their responses as to whether they liked having ADHD or being autistic varied more, with 29% agreeing “somewhat” or “fully” with their diagnosis. This suggests that “diagnosis agreement” and “diagnosis liking” reflect differential processes in diagnosis identity (i.e., cognitive vs emotional acceptance). Thus, recognizing a neurodevelopmental diagnosis as accurate does not necessarily imply embracing it as a positive part of one’s identity. This distinction fits well with previous research on illness representations, suggesting that cognitive recognition and emotional integration are separable processes, each with distinct implications for adjustment and other psychosocial outcomes (5).

The second research question concerned masking. Masking is a common way to adapt to societal structures in individuals with neurodevelopmental conditions (31, 32), and our study revealed that masking is not a fixed trait, but rather a situational strategy. Participants reported that they masked their personality more in school/at work and in their family, but much less so with neurodivergent peers. Individuals may feel safer to be authentic among peers but feel pressure to conform in other scenarios. While neurodivergent peer settings seemed to support authenticity, family and school/work contexts likely add more external pressures or stigma (29). Hence, these findings extend prior research on social camouflaging by adding the importance of considering contextual factors that trigger masking behaviors (33). Interestingly, although diagnostic group comparisons showed some differences with regard to masking, effect sizes were small. Thus, although masking has mainly been described among individuals with ASD, this finding supports the growing evidence that masking behaviors are also relatively common among individuals with ADHD (29). In sum, our findings show that diagnosis acceptance and masking are dynamic and context-sensitive processes that shape the experience of neurodivergent individuals across diagnostic groups.

Regarding the third research question, participants identified a broad range of diagnosis benefits, but also challenges. Benefits were varied, with cognitive benefits such as “seeing things my own way” and “drive to find things out” being the most commonly endorsed items. Social or communal benefits (e.g., “community with others with my diagnosis”) were not commonly endorsed. This points to a lack of a sense of community or access to peer networks for neurodivergent individuals, which prior research on autistic community connectedness highlighted as key to people’s wellbeing (41).

The benefits most strongly associated with quality of life were “positive energy” and “drive to find things out.” Of note is that these two types of benefits are usually connected with two of the ADHD symptoms (i.e., hyperactivity and impulsivity) and not the third (i.e., inattention). This may have important clinical implications as group-based treatments for ADHD, as they often include information about how to identify strengths/benefits. However, if the benefits most strongly associated with quality of life are only present among individuals with the combined or hyperactive/impulsive presentation of ADHD, this points to the need for group treatments only targeting the inattentive presentation (42).

Challenges related to having ADHD and/or ASD were more universally endorsed than benefits, particularly the items “mental exhaustion” and “being misunderstood.” This finding aligns with the minority stress model (8), which implies that chronic exposure to stigma can lead to heightened stress and poorer mental health outcomes, and this model has also been shown to be relevant for neurodivergent individuals (6). When comparing participants with ADHD and/or ASD, individuals with both conditions were somewhat more inclined to endorse both more benefits and more challenges, which is possibly due to additive effects of dual diagnoses. In summary, while participants often recognized diagnosis challenges, it is also important to acknowledge that a substantial proportion of individuals with ADHD and/or ASD also report benefits.

The fourth research question focused on how diagnosis liking, masking, benefits, and challenges are related to quality of life. Overall, quality of life was positively linked to liking one’s diagnosis and perceived benefits but negatively associated with masking behaviors and perceived challenges. The ADHD group scored significantly higher in the quality of life than the ASD group. The regression confirmed that each variable independently contributed to quality of life, with perceived benefits showing the strongest association. These patterns align with the ACT framework, in which acceptance (agreement/liking), values-based living (perceiving benefits), and experiential avoidance (masking) jointly shape psychological flexibility, a central determinant of quality of life (36). Perceived challenges, in turn, may represent barriers that undermine acceptance and valued action, thereby contributing to lower quality of life.

Notably, liking one’s diagnosis was associated with diagnosis benefits, but surprisingly, the correlation was substantially weaker with the number of challenges they perceived. This finding reinforces that benefits and challenges coexist and that individuals with neurodevelopmental diagnoses may maintain a realistic awareness of challenges while still embracing their diagnosis (15, 18). Moreover, masking appeared to be highly context sensitive. While masking in school/work and family settings was associated with greater perceived challenges, masking with neurodivergent peers was negatively related to perceived benefits and only weakly related to challenges. This supports the view that understanding masking within a broader context is crucial (29, 33), and emphasizes the need for supportive environments that build a positive diagnosis identity, thereby reducing masking demands.

Finally, our sensitivity analyses showed that participants with and without medication did not differ significantly regarding any of the variables included in the study. However, comorbid diagnoses (both when examined as mood disorders and any comorbid disorder) did have a significant effect, although effect sizes were mostly small. Regarding medication, our finding is in line with a review showing that most studies on adult ADHD do not show a medication effect on quality of life or daily functioning (43). Regarding comorbidity, our results are in line with previous research showing that individuals with either ADHD and/or ASD who also have additional comorbid diagnoses report lower quality of life, more challenges, and higher burden in daily life (44–46). However, there is still a need for more studies examining how comorbidity (e.g., mood disorders) influences quality of life and potential benefits among adults with ADHD and/or ASD.

### 4.1 Strengths and limitations

The present study had several strengths and limitations. Regarding strengths, there is still a shortage of studies on ADHD and ASD in adulthood compared to childhood, and most studies have focused on deficits and not included potential benefits. The studies that have focused on more positive aspects of having ADHD and/or ASD have used a qualitative approach, and this is, to our knowledge, the largest study among the few quantitative studies available. Moreover, unlike many studies that focus exclusively on either benefits or challenges, this study provides a balanced perspective by examining both positive aspects and difficulties associated with having a neurodevelopmental diagnosis. Finally, it has been emphasized (16) that we need to relate positive aspects to clinically relevant outcomes, and the present study therefore adds valuable new information by showing that liking one’s diagnosis and perceived benefits are associated with quality of life.

Regarding limitations, a major issue is that the representativeness of our sample can be questioned. Females were overrepresented (i.e., 79%), diagnoses were most often not received until adulthood, and a relatively large proportion of the sample (71%) was in employment or studying. Thus, findings may not generalize to individuals diagnosed earlier in life, who often have more severe impairment (39). Another important limitation is that our large sample size prevented us from conducting diagnostic interviews within the study to verify the diagnoses. Third, this dataset was part of a larger study, which meant we had to keep the number of questions for each domain low and therefore could not include longer, validated scales in our survey. Thus, future studies need to investigate if the results of the present study can be replicated using more psychometrically validated measures, and there is also a need for longitudinal studies within this area. Additionally, our sensitivity analyses, which revealed significant effects of comorbidity, suggest that further research is needed in this area.

## 5 Conclusions

This large-scale study provides insights into how adults with ADHD and/or ASD perceive their diagnoses and how this is related to quality of life. We found that while most participants agreed with their diagnosis, more variation (mostly within rather than between diagnostic groups) was observed for liking to have ADHD and/or ASD. Masking behaviors were common and were dependent on the context. Importantly, while participants recognized benefits but also challenges related to their diagnosis, the total amount of perceived benefits emerged was most strongly associated with quality of life. Thus, diagnosis benefits are related to important real-life outcomes, highlighting the value of clinical interventions and psychoeducational programs that go beyond identifying deficits and also acknowledge strengths. It has also been argued that knowledge about strengths can be beneficial for making sound career choices, thereby potentially increasing educational and occupational success (47). A more balanced view on neurodevelopmental diagnoses that includes strengths can also facilitate support and reduce stigma for individuals with neurodevelopmental disorders. It has also been shown that individuals with ADHD themselves prefer coaching that uses a strength-based approach compared with more traditional symptom-based coaching (48). Conclusively, increased knowledge of the strengths related to ADHD and ASD can potentially have important clinical implications.

## Data Availability

The raw data supporting the conclusions of this article will be made available by the authors, without undue reservation.
