# Supplementary material for: Diagnosis acceptance, masking, and perceived benefits and challenges in adults with ADHD and ASD: associations with quality of life
Source: Front Psychiatry. 2025 Oct 15;16:1668780. doi: 10.3389/fpsyt.2025.1668780 (PMC12568611; doi:10.3389/fpsyt.2025.1668780)
Supplement: Supplementary file 1 [file DataSheet1.pdf]

Supplementary Material

Supplementary Table 1. Participant Characteristics

	ADHD (N = 803)	ASD (N = 158)	ADHD+ASD (N = 95)
Age, years (SD)	28 (4.8)	26 (7.3)	26 (5.4)
Sex (% female)	82.6	61.8	70.4
Marital Status (%)			
Single	36.2	74.1	52.6
Married/ partnership/ cohabiting	61.2	25.3	44.2
Separated / divorced	2.4	0.6	3.2
Employment (%)			
Employed (full-time)	43.2	23.4	15.8
Employed (part-time)	10.2	10.1	7.4
Unemployed	7.0	8.2	5.3
School student/ Student	21.2	24.7	28.4
Disability pension	12.5	25.3	30.5
Other	5.7	8.2	12.7
Age at Diagnosis (%)			
Childhood	12.7	21.7	31.0
Adolescence	16.6	22.4	19.5
Adulthood	70.7	55.9	49.4
Time since diagnosis, years (SD)	6.08 (6.85)	6.35 (8.07)	8.15 (7.63)
Unofficial Diagnoses (%)			
One other	30.9	41.8	6.3
Two others	1.2	1.9	0.0
Medication (%)	82.2	64.8	66.7
Comorbidities (%)			
Anxiety	58.0	56.3	48.4
Depression	60.0	57.6	52.6
Eating Disorder	20.5	16.5	20.0
PTSD [a]	20.5	14.6	27.4
Dyslexia/Dyspraxia/Dyscalculia	15.6	10.8	23.2
Intellectual disability	0.4	3.2	2.1
Sound Sensitivity	22.0	29.7	41.1
Other sensory difficulties	12.3	24.1	30.5
Exhaustion Disorder	39.5	29.7	41.1
Other mental condition [b]	30.3	27.8	38.9
Somatic/neurological conditions [c]	38.7	36.1	45.3

Note. [a] post-traumatic stress disorder, [b] obsessive-compulsive disorder, body-focused repetitive behaviors, bipolar disorder, schizophrenia, [c] Epilepsy, Allergies, Digestive problems (IBS/IBD), cerebral palsy, ME/CFS/Fibromyalgia

Supplementary Table 2. Sex Differences: means, standard deviations, and t-test statistics

	Males n = 212	Females n = 787			
	M(SD)	M(SD)	*t*(997)	*p*	Cohen's d
Liking	2.68 (1.21)	2.63 (1.21)	0.50	n.s.	0.04
Masking: school	3.54 (1.43)	4.23 (0.97)	-8.19	< .001	0.64
Masking: family	2.85 (1.47)	3.36 (1.35)	-4.81	< .001	0.37
Masking: peers	2.24 (1.22)	2.25 (1.23)	-0.08	n.s.	0.01
Benefits	3.44 (1.98)	3.70 (2.05)	-1.64	n.s.	0.13
Challenges	3.29 (1.56)	3.79 (1.48)	-4.32	< .001	0.33
Quality of Life	5.48 (2.16)	5.49 (2.02)	-0.08	n.s.	0.01

Supplementary Table 3. *Diagnostic Group Differences: categorical differences in diagnosis benefits and challenges*

	ADHD Group (1) n = 803	ASD Group (2) n = 158	ADHD+ASD group (3) n = 95	Total N = 1,056			
	N (%)	*N* (%)	*N* (%)	*N* (%)	χ^2	*Cramér's V*	Post-hoc [a]
Benefits							
Seeing things my own way	519 (64.6)	95 (60.1)	76 (80.0)	690 (65.3)	11.09**	.10	3 > 1, 2
Sense of justice	430 (53.5)	83 (52.5)	60 (63.2)	573 (54.3)	3.38	.06	n.s.
Community with others with the diagnosis	215 (26.8)	41 (25.9)	36 (37.9)	292 (27.7)	5.52	.07	n.s.
Attention to details	397 (49.4)	97 (61.4)	61 (64.2)	555 (52.6)	13.25**	.11	2, 3 > 1
Honesty	356 (44.3)	86 (54.4)	59 (62.1)	501 (47.4)	14.40***	.12	2, 3 > 1
Distinctive features	275 (34.2)	31 (19.6)	33 (34.7)	339 (32.1)	13.29**	.11	1, 3 > 2
Positive energy	232 (28.9)	17 (10.8)	19 (20.0)	268 (25.4)	24.51***	.15	1 > 2
Drive to find things out	512 (63.8)	80 (50.6)	60 (63.2)	652 (61.7)	9.72**	.10	1 > 2
Challenges							
Physically tiring	489 (60.9)	71 (44.9)	58 (61.1)	618 (58.5)	14.13***	.12	1, 3 > 2
Mentally exhausting	766 (95.4)	141 (89.2)	87 (91.6)	994 (94.1)	10.27**	.10	1 > 2
Differential treatment	227 (28.3)	62 (39.2)	55 (57.9)	344 (32.6)	37.70***	.19	3 > 2 > 1
Being misunderstood	669 (83.3)	130 (82.3)	87 (91.6)	886 (83.9)	4.66	.07	n.s.
Being disrespected	290 (36.1)	73 (46.2)	58 (61.1)	421 (39.9)	25.15***	.15	3 > 2 > 1
Criticism of things related to the diagnosis	507 (63.1)	88 (55.7)	64 (67.4)	659 (62.4)	4.21	.06	n.s.

* $p < .05$, ** $p < .01$, *** $p < .001$

Supplementary Table 4. *Summary of Multiple Regression Analysis*

				95% CI		
	ß	*B*	*SE*	*LL*	*UL*	*p*
Liking	.13	.22	.05	.12	.33	<.001
Masking						
School/Work	-.13	-.24	.06	-.36	-.11	<.001
Family	-.13	-.20	.05	-.30	-.10	<.001
Neurodivergent Peers	-.08	-.13	.05	-.23	-.03	.014
Number of Benefits	.22	.22	.03	.15	.29	<.001
Number of Challenges	-.13	-.17	.04	-.26	-.09	<.001

Notes. N = 940. CI = confidence interval. LL = lower level. UL = upper level
